# Combination Therapy with Rituximab, Tofacitinib and Pirfenidone in a Patient with Rapid Progressive Interstitial Lung Disease (RP-ILD) Due to MDA5 Antibody-Associated Dermatomyositis: A Case Report

**DOI:** 10.3390/medicina57121358

**Published:** 2021-12-13

**Authors:** Tsai-Hung Yen, Chih-Wei Tseng, Kao-Lun Wang, Pin-Kuei Fu

**Affiliations:** 1 Division of Allergy, Immunology and Rheumatology, Department of Internal Medicine, Taichung Veterans General Hospital, Taichung 40705, Taiwan; mi552296@gmail.com (T.-H.Y.); cwtseng@vghtc.gov.tw (C.-W.T.); 2 Department of Radiology, Taichung Veterans General Hospital, Taichung 40705, Taiwan; y0107124@yahoo.com.tw; 3 Department of Critical Care Medicine, Taichung Veterans General Hospital, Taichung 40705, Taiwan; 4 Integrated Care Center of Interstitial Lung Disease, Taichung Veterans General Hospital, Taichung 40705, Taiwan

**Keywords:** MDA-5, RP-ILD, rituximab, tofacitinib, pirfenidone

## Abstract

Anti-melanoma differentiation-associated protein 5 (MDA5)-positive rapidly progressive interstitial lung disease (RP-ILD) is associated with poor prognosis, and the most effective therapeutic intervention has not been established. Herein we report a case of a 45-year-old female patient who presented with myalgia, Gottron’s papules with ulceration, and dyspnea on exertion which became aggravated within weeks. Laboratory examination and electromyography confirmed myopathy changes, and a survey of myositis-specific antibodies was strongly positive for anti-MDA5 antibody. High-resolution chest tomography suggested organizing pneumonia with rapidly progressive changes within the first month after diagnosis of the disease. Anti-MDA5-associated dermatomyositis with RP-ILD was diagnosed. Following combination therapy with rituximab, tofacitinib and pirfenidone, clinical symptoms, including cutaneous manifestation, respiratory conditions and radiographic changes, showed significant and sustainable improvement. To our knowledge, this is the first reported case of anti-MDA5-associated dermatomyositis with RP-ILD successfully treated with the combination of rituximab, tofacitinib, and pirfenidone.

## 1. Introduction

Anti-melanoma differentiation-associated gene 5 (MDA5) antibody-associated interstitial lung disease (ILD) has been recognized as an important entity in patients with dermatomyositis-associated interstitial lung disease, especially rapidly progressive interstitial lung disease (RP-ILD) [1]. Anti-MDA5 antibody, first described in a Japanese cohort of patients with inflammatory myositis by Sato et al. in 2005, targets the autoantigen MDA5, a cytoplasmic RIG-I-like receptor, and has the ability to enhance innate immunity by suppressing viral particle replication [2]. The unique manifestation of anti-MDA5-positive dermatomyositis, including distinct mucocutaneous features, clinically amyopathic or hypomyopathic changes, and most importantly, rapidly progressive interstitial lung disease, are now well acknowledged, but the expression level of anti-MDA5 and its association with the prognosis of the disease remain unclear [3]. 

Anti-MDA5-positive RP-ILD was found to be associated with a poor prognosis, and the initial combination use of immunosuppressive agents was shown to yield a significantly better survival rate in comparison to step-up therapy [1,4]. Most combination regimens include a glucocorticoid, a calcineurin inhibitor, cyclophosphamide, and rituximab, though the regimen with the best efficacy has not yet been determined [4]. Tofacitinib, a JAK1/3 inhibitor which modulates various cytokines critical in inflammatory and immune responses, was also demonstrated recently to be effective in treating anti-MDA5-positive ILD [5]. In addition to the use of an immunosuppressant, anti-fibrotic agents have also been proposed to be potentially beneficial in a subgroup of patients [6]. 

In the present case, anti-MDA5-associated RP-ILD was successfully treated using the immunosuppressive agents rituximab and tofacitinib in combination with pirfenidone, which resulted in near-complete resolution of pulmonary infiltrates.

## 2. Case Presentation

A 45-year-old female patient with past history of hypertension for 2 years presented to our emergency room with the chief complaint of dyspnea on exertion and low-grade fever for 2 weeks. She also mentioned insidious onset of muscle soreness of the anterior thighs for one month, which was followed by the appearance of itchy rashes over the knuckles of both hands and the sun-exposed area of the anterior chest. She was first diagnosed with community-acquired pneumonia a week before coming to our emergency room, but empirical antibiotics had failed to improve her symptoms. Physical examination at admission revealed a temperature of 38.1 °C, a pulse rate of 104 beats per minute, a respiratory rate of 21 breaths per minute, pulse oximeter Sp02 88% under ambient air, inspiratory rales over bilateral lower lung, Gottron papules at bilateral PIP and MCP joints with ulceration over the right MCP3 joint, V sign with cutaneous ulceration, periungual erythema, and normal muscle power. 

**Figure 1:**
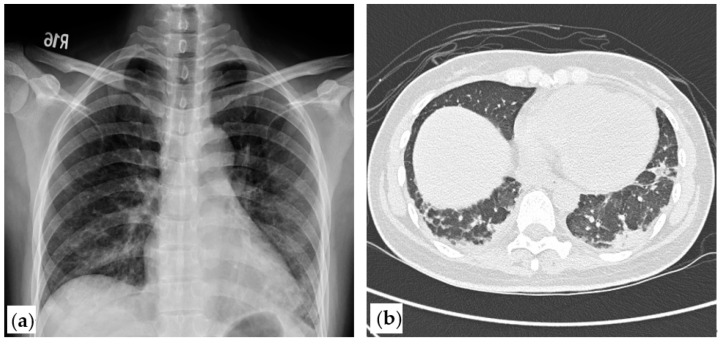
Chest X-ray and high-resolution computed tomography (HRCT) of the patient. (**a**). Chest X-ray showed interstitial infiltration of bilateral lower lungs. (**b**). High-resolution computed tomography (HRCT) at week 0 showed subpleural consolidation with interlobular septal thickening.

Laboratory examination revealed a total leukocyte count of 4640/μL, hemoglobin concentration of 10.9 g/dL, and platelet count of 262,000/μL. Her ALT was 100 U/L, AST was 125 U/L, serum total bilirubin was 0.5 mg/dL, lactate dehydrogenase was 422 U/L, creatine kinase (CK) was 264 U/L, creatinine was 0.52 mg/dL, and thyroid function test was normal. In addition, autoimmune tests showed anti-nuclear antibody 1:80 (equivocal), negative anti-SSA/SSB, negative anti-Jo-1, and a strongly positive anti-MDA5 detected by a commercial line-blot immunoassay kit (EUROIMMUN-DL 1530-1601-4 G). Nerve conduction velocity and electromyography disclosed mild myopathic changes indicated by BSAPPs over bilateral iliopsoas muscles and deltoid muscles. Chest X-ray showed increased interstitial infiltration over bilateral lower lung fields. High-resolution computed tomography (HRCT) showed subpleural consolidation with interstitial thickening of bilateral lungs with lower lung predominance (Figure 1). Anti-MDA5-associated DM with ILD was diagnosed.

**Figure 2:**
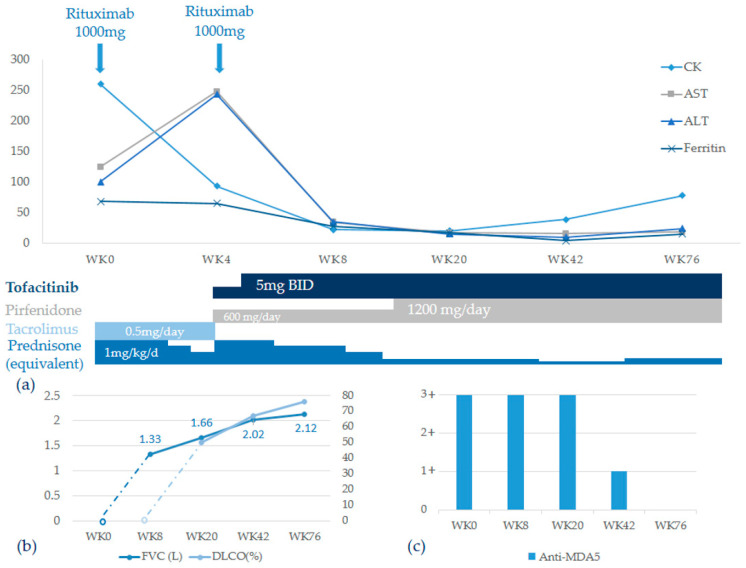
Clinical course of treatment, lung function, and anti-MDA serum level at follow-up. (**a**). Combination therapy resulted in early improvements of muscle enzymes, which remained stable throughout the disease course. (**b**). Both FVC and DLCO normalized gradually under therapy. (**c**). Levels of anti-MDA5 declined since week 42 and turned negative on week 76.

**Figure 3:**
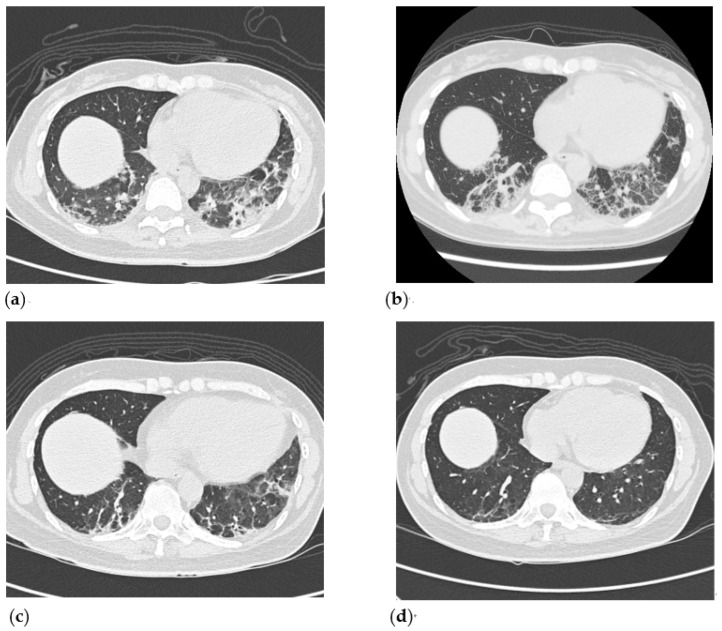
Serial HRCT follow-up (axial view) on week 4, week 8, week 20, and week 42. (**a**). HRCT arranged on week 4 showed increased peribronchial and subpleural consolidation and ground glass opacities (GGO) over the left lower lung. (**b**). HRCT on week 8 revealed partial resolution of subpleural consolidation over the left lower lobe. However, interlobular septal thickening and a peri-lobular pattern at the subpleural area of bilateral lower lungs were noted on the HRCT image. (**c**). HRCT on week 20 demonstrated obvious resolution of subpleural consolidation and GGO over bilateral lower lung fields. (**d**) HRCT on week 42 disclosed regressive change of GGO lesion in bilateral lungs, and only a small fibrotic band existed in bilateral lower lung fields.

We started the treatment with an equivalent dosage of 0.4 mg/kg/day oral prednisone, with intravenous rituximab at a dose of 1000 mg and tacrolimus at a dose of 0.5 mg/day. Fever subsided following the treatment, and her cutaneous lesions and dyspnea improved one week later. However, skin rashes recurred at week 4 and dyspnea on exertion also worsened. Laboratory examination during readmission revealed elevated liver enzymes (ALT 233 U/L, AST 248 U/L) and lower CK levels (93 U/L) (Figure 2a). Progression of interstitial lung disease was evident on HRCT, which showed increased peribronchial and subpleural consolidation with ground glass opacities in bilateral lungs (Figure 3a). A multidisciplinary team comprising a rheumatologist, a pulmonologist, and a radiologist was created as the disease progressed rapidly, and a diagnosis of organizing pneumonia related to anti-MDA5-positive RP-ILD was reached by the team. Due to RP-ILD, corticosteroid was titrated from 0.4 mg/kg/day to 0.8 mg/kg/day, and the second dose of rituximab was given 3 weeks after the first dose. Additionally, we shifted tacrolimus to tofacitinib with a dosage of 5 mg twice daily. At the same time, the anti-fibrotic agent pirfenidone was started with a dosage of 600 mg per day in combination with the immunosuppressive agents due to unsatisfactory treatment response. Respiratory condition and rashes improved slowly, and the patient still depended on home oxygen therapy upon discharge. The dosage of pirfenidone was titrated to 1200 mg per day as the patient could tolerate it well. It was not until week 20 that the patient was able to do without oxygen therapy, and repeated HRCT showed regressive changes of the organizing pneumonia with partial resolution of subpleural consolidative patches (Figure 3c). The corticosteroid was successfully tapered from 0.8 mg/kg/day to 0.1 mg/kg/day. The residual subpleural interstitial thickening and ground glass opacities on HRCT showed substantial improvement and near-complete resolution of pulmonary infiltrates at week 42 (Figure 3d).

Lung function testing, including forced vital capacity and diffusing capacity of carbon monoxide, was not tolerable due to dyspnea initially until week 20 post-therapy, and it revealed restrictive lung disease with moderately impaired gas exchange. Both forced vital capacity and diffusing capacity of carbon monoxide also improved markedly under serial follow-up in concordance with the findings on HRCT (Figure 2b). The titer of anti-MDA5 declined at week 42 and turned negative at week 76 (Figure 2c). The clinical symptoms and muscle enzymes of the patient remained stable throughout the follow-up of 76 weeks. 

## 3. Discussion

Anti-MDA5-positive RP-ILD was associated with poor prognosis and the 90-day survival rate was only 66.7% despite aggressive conventional treatments [1]. The treatment options for RP-ILD associated with anti-MDA5-positive DM are not well established, and combination therapy with various immunosuppressant agents has been advocated [4]. Rituximab, a chimeric monoclonal antibody against CD-20, was demonstrated in small retrospective studies to be effective in the treatment of anti-MDA5 positive RP-ILD. In the Ge Y retrospective survey of 11 patients with MDA-5-positive RP-ILD, rituximab yielded a 73% response rate, while in a similar study conducted by So H. involving 4 patients with refractory interstitial lung diseases, the treatment response of rituximab was as high as 100% [7,8]. Although the evidence supporting the use of rituximab in MDA-5-associated DM is limited to case series, a large, randomized control trial (RIM trial) demonstrated that 83% of patients with refractory myositis responded to rituximab, and the post hoc analysis suggested positive autoantibody expression in patients was associated with a trend of lung function improvement [9]. Interestingly, in our patient, rituximab therapy produced a gradual, sustained effect, rather than a rapid response. The obvious resolution of lung infiltrates only appeared at week 20, and the level of anti-MDA5 antibody also remained strongly positive within 4 months after treatment. The phenomenon of delayed clinical improvement of rituximab was also observed in the RIM trial, in which the primary endpoint was not met at week 8, and clinical responsiveness to rituximab was not evident until week 42 [9].

The treatment response of our patient was further enhanced by the combination therapy of tofacitinib, a selective JAK1/3 inhibitor which blocks multiple aspects of cytokine signaling. The successful use of tofacitinib in MDA-5-associated ILD was first reported by Kurasawa K., who investigated 5 Japanese patients with refractory diseases [10]. The survival benefit of tofacitinib was further illustrated in a prospective study of 29 patients in China, which found that JAK inhibitor therapy in the early stage of the disease before decompensated pulmonary function resulted in a better 6-month survival rate compared to the historical control group [5]. In contrast to rituximab, the rapidity of the therapeutic response to JAK inhibitors has been shown in patients with rheumatoid arthritis, and the response could be achieved as early as two weeks [11]. Therefore, incorporating tofacitinib with rituximab may reasonably fill the gap between the initiation of rituximab and the onset of its therapeutic effect. To our knowledge, the present study is the first reported case of anti-MDA5-associated RP-ILD treated successfully with a combination of rituximab and tofacitinib in the literature. 

Progressive fibrosing ILD represents a distinct phenotype of ILD with a poor prognosis, and connective tissue disease-related ILD (CTD-ILD) is an important etiological component The mechanism of fibrosis related to CTD-ILD is not fully understood but is known to involve innate and adaptive immunity contributing to the development of fibrosis. As Spagnolo et al. pointed out, the progressive fibrosing phenotype of ILD may arise from common underlying pathophysiological mechanism of fibrosis [12]. Two of the anti-fibrotic agents, nintedanib and pirfenidone, which have been approved for idiopathic pulmonary fibrosis (the archetypal ILD with a progressive phenotype) were now both tested in CTD-ILD [12,13]. Early success in slowing lung function decline was achieved in the phase III trial of nintedanib involving patients with scleroderma-related ILD [13]. On the other hand, in an early terminated phase II study, pirfenidone was shown to reduce decline in FVC% predicted over 48 weeks compared to placebo in patients with progressive forms of fibrotic ILD, including 28% patients with CTD-ILD [14]. In addition to an anti-fibrotic effect, pirfenidone possesses multiple effects which include not only the regulation of profibrotic cytokine cascades, inhibition of fibroblast proliferation, and synthesis of collagen, but also the downregulation of inflammation [15]. For the treatment of patients with anti-MDA-5-associated RP-ILD, although it was not tested in a large prospective trial, pirfenidone was shown to improve survival rate, especially in those with subacute disease (disease duration 3 to 6 months) in a single retrospective study [6]. In our patient, we decided to add on pirfenidone at the time when disease progression was not ameliorated by the initial treatment of immunosuppressive agents. The pirfenidone was used in combination with rituximab and tofacitinib. Radiographic improvement at week 20 was seen predominantly in peripheral consolidations, but residual fibrotic change, including reticulation and traction bronchiectasis, remained prominent, though it did resolve gradually and substantially during follow up. The resolution of both fibrosis and inflammatory infiltrates could mainly be attributed to the effect of immunosuppressants, and to what extent the use of pirfenidone provided beneficial was unclear. The result of an ongoing phase III trial (NCT03221257) assessing the combined use of mycophenolate mofetil and pirfenidone on systemic sclerosis-related ILD will determine whether the antifibrotic effects of pirfenidone will complement the anti-inflammatory effects of immunosuppressants. 

Pulmonary function tests and HRCT are considered to be essential both at baseline and during follow-up for the diagnosis of CTD-ILD and for early detection of deterioration [16]. The progression of anti-MDA5-positive RP-ILD may occur within three months; in our case, it occurred over one month. The immunomodulators were intensified in a timely manner in our case, as HRCT confirmed progression, and the treatment response could also be supported by the simultaneous improvement of both radiographic findings and parameters measured by spirometry. To predict the prognosis and assess disease activity, our findings imply that it may be mandatory for the treating clinician to incorporate symptoms, lung function testing, radiographic modalities, and serum biomarkers, if possible. Several biomarkers have been proposed as indicators for disease activity, including ferritin, IL-18, KL-6, and anti-MDA5 levels [17]. The level of ferritin in our patient was not consistent with rapid progressive lung disease, though it may still serve as a prognostic factor. Gono T. et al. reported that a baseline ferritin level of less than 500 ng/mL was associated with an excellent 60-day survival rate [18]. In the same retrospective study of 27 Japanese patients, anti-MDA5 titer was significantly lower after treatment than on admission in the living subset, which suggests its utility in monitoring disease activity. Of note, the clearance of autoantibody of our patient lagged the clinical improvement, and the pathogenic role of anti-MDA5 antibody is not well understood. A recent study showed circulating neutrophil extracellular traps (NETs) were increased in inflammatory myopathy, and serum anti-MDA5 correlated with circulating and tissue NETs, even directly enhancing NET formation [19]. Despite the uncertainty, obtaining serial measurements of biomarkers may still be beneficial as part of a multidirectional evaluation of this rapidly progressive disease.

## 4. Conclusions

The present study is the first reported case of anti-MDA5-associated RP-ILD successfully treated with a combination of intensive immunosuppressive agents including rituximab, tofacitinib, and pirfenidone. This case emphasizes that the incorporation of rituximab and tofacitinib resulted in both clinical remission and serological response with loss of anti-MDA5 autoantibody. Whether the favorable outcome of our patient could be attributed to the addition of pirfenidone or mainly to the effect of the immunosuppressants is unclear. Prospective studies to evaluate the role of pirfenidone in combination with immunosuppressive agents in treating RP-ILD are warranted in the future.
